# Discovery and validation of mucosal TNF expression combined with histological score - a biomarker for personalized treatment in ulcerative colitis

**DOI:** 10.1186/s12876-020-01447-0

**Published:** 2020-10-02

**Authors:** Jon R. Florholmen, Kay-Martin Johnsen, Renate Meyer, Trine Olsen, Øystein K. Moe, Petter Tandberg, Mona D. Gundersen, Jan-Magnus Kvamme, Knut Johnsen, Terje Løitegård, Gabriele Raschpichler, Cecilia Vold, Sveinung W. Sørbye, Rasmus Goll

**Affiliations:** 1 Research Group of Gastroenterology and Nutrition, Department of Clinical Medicine, University of Tromsø, Tromsø, Norway; 2 Department of Gastroenterology, Division of Internal Medicine, University Hospital of North Norway, Tromsø, Norway; 3 Department of Internal Medicine, Vestre Viken Hospital, Hønefoss, Norway; 4 Department of Internal Medicine, Hammerfest Hospital, Hammerfest, Norway; 5 Department of Gastroenterology, Vestre Viken Hospital, Drammen, Norway; 6 Department of Internal Medicine, Kirkenes, Norway; 7 Department of Gastroenterology, Nordland Hospital, Bodø, Norway; 8 Department of Pathology, University Hospital of North Norway, Tromsø, Norway

**Keywords:** antiTNF, Calprotectin, Cytokines, Diagnostic odds ratio, Robarts histopathology index

## Abstract

**Background:**

There are no accurate markers that can predict clinical outcome in ulcerative colitis at time of diagnosis. The aim of this study was to explore a comprehensive data set to identify and validate predictors of clinical outcome in the first year following diagnosis.

**Methods:**

Treatment naive-patients with ulcerative colitis were included at time of initial diagnosis from 2004 to 2014, followed by a validation study from 2014 to 2018. Patients were treated according to clinical guidelines following a standard step-up regime. Patients were categorized according to the treatment level necessary to achieve clinical remission: mild, moderate and severe. The biopsies were assessed by Robarts histopathology index (RHI) and TNF gene transcripts.

**Results:**

We included 66 patients in the calibration cohort and 89 patients in the validation. Mucosal TNF transcripts showed high test reliability for predicting severe outcome in UC. When combined with histological activity (RHI) scores the test improved its diagnostic reliability. Based on the cut-off values of mucosal TNF and RHI scores from the calibration cohort, the combined test had still high reliability in the validation cohort (specificity 0.99, sensitivity 0.44, PPV 0.89, NPV 0.87) and a diagnostic odds-ratio (DOR) of 54.

**Conclusions:**

The combined test using TNF transcript and histological score at debut of UC can predict severe outcome and the need for anti-TNF therapy with a high level of precision. These validated data may be of great clinical utility and contribute to a personalized medical approach with the possibility of top-down treatment for selected patients.

## Background

Ulcerative colitis (UC) is one of the two main disease entities of inflammatory bowel disease (IBD). UC is a chronic inflammatory disease believed to result from a dysregulated immune response caused by a combination of environmental and genetic factors causing loss of immunotolerance in the gut [1]. Many patients experience severe outcomes of disease with significant reduction in quality of life. The need for surgery is reported in 8 and 9.7% after 5 and 11 years, respectively [2, 3].

Definitions of clinical outcomes and prognosis in IBD are poorly defined, with little agreement on primary and secondary endpoints [4]. The IBSEN study is one of the most well-known prospective studies on clinical outcomes in UC, where the patients were divided into 4 predefined patterns of disease [2]. In a recently published review, the extent of disease and high disease activity were predictors of a more severe progression of disease [5].

The Montréal guidelines classify UC disease activity into four categories; clinical remission, mild, moderate and severe disease [6, 7]. Different guidelines for medical and surgical treatment are available for both UC and CD in Europe and America, European Crohn’s and Colitis Organization (ECCO) guidelines and American Gastroenterological Association (AGA) clinical care pathway repectively [8, 9]. Danese et al. have created a modified algorithm with a medical step-up approach for the treatment of UC with the goal of achieving clinical remission [10]. In short, 5-ASA and local steroids are used in mild disease, with additional oral steroids, immunosuppressive and biological therapy in moderate to severe disease, consecutively. In contrast, a so-called top-down therapy has previously been documented to induce long term clinical remission of Crohn’s disease [11].

From a clinical point of view, there is a need to find good predictive markers at onset of disease that enables clinicians to individually tailor therapy. There is an increasing interest for a biomarker approach. In various diseases, such as breast cancer, four gene subtypes of human epidermal growth factor receptor 2 (*HER2)* forms the basis of a molecular reclassification of disease according to risk factors [12]. Although there are an increasing number of reports and reviews for clinical and biochemical biomarkers at onset of disease, none have been able to predict future clinical outcome with great certainty [13–18]. In our research group we have published reports on mucosal transcript levels of tumor necrosis factor (TNF) as a biomarker for response to and when to stop anti-TNF thereapy [19–21], However, most of the studies are of retrospective design and there is a lack of validated studies of prognostic biomarkers to predict the clinical outcome in IBD with high reliability. Moreover, a personalized therapy approach initiated at the time of disease diagnosis, may have an impact on the natural course of IBD. This is so far unsettled due to the lack of long- term studies [22–24].

There is increasing knowledge of the pathophysiological events mediating the mucosal inflammation in IBD including cytokine and chemokine responses [25, 26]. So far there are few reports on how these crucial mediators can be used as biomarkers [19–22]. Therefore, the aims of this study were, first, based on a calibration cohort of newly diagnosed patients with ulcerative colitis from 2004 to 2014, to discover potential clinical, biochemical, histological and mucosal gene transcripts to predict 1 year level of treatment to obtain remission. Second, to validate these parameters in a cohort study from 2014 to 2018.

## Methods

The main goal of the study was to detect and validate potential predictors of treatment level 1 year after disease onset of UC. In principle, to do a proper validation of a predictor(s) it is general accepted that this should be a two-step procedure. First, we have to study a calibration (discover) cohort, followed by a study of a validation cohort to validate the candidate predictors from the discovery study. Inclusion criteria for both the discovery and validation cohort were patients with newly diagnosed, treatment- naive UC aged ≥18 years. Patients were excluded if they were lost to follow in the first year after diagnosis, patients with severe medical disease other than UC, pregnancy and lactation; and patients who first were diagnosed UC but later developed an indeterminate form of IBD.

In addition to the UC patients with newly diagnosed, treatment-naïve disease, a group of healthy subjects performing a cancer screening examination with no clinical, endoscopic or histological signs of intestinal disease were included as controls.

### Cohorts examined

#### Calibration cohort

Patients attending the Gastrointestinal Unit at the University Hospital of North Norway, Tromsø, Norway, were recruited from the project *Immunopathogenesis in inflammatory bowel disease* in the time period January 2004 –March 2014. *Validation cohort:* Patients were recruited in the time period March 2014 –March 2018 attending 6 clinical centers in Norway (Gastrointestinal units at the hospitals of Kirkenes, Hammerfest, University Hospital North Norway, Tromsø, Bodø, Vestre Viken (Ringerike and Drammen)) as a part of an ongoing prospective study - *Advanced Study of Inflammatory Bowel disease* (ASIB- study).

### Diagnosis, clinical grading and clinical outcome after 1 year

The clinical grading of UC was based on evaluation of clinical activity at 1 year. The biopsies were histologically assessed by an experienced pathologist (SWS) using Robarts histopathology index (RHI) score [27].

**Fig. 1:**
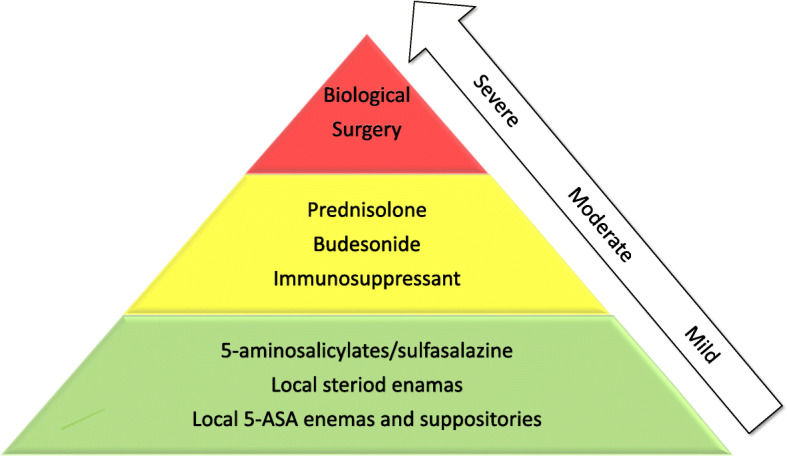
In this and the following figures data from patients with ulcerative colitis at debut of disease are grouped after 1-year treatment level outcome, Step-up algorithm according to clinical treatment outcomes (mild, moderate, severe). Modified after Danese et al., see ref. [8]

The clinical outcomes of UC are based on the required treatment level to obtain disease remission, using the step-up algorithm guidelines ECCO and the three levels proposed by Danese et al. [10, 28] In this study we used three disease outcome levels after 1 year; mild, moderate and severe. These outcomes were defined by the treatment level needed for clinical remission; 5-ASA per oral or local (mild), need of oral steroids and/or thiopurines (moderate) and need of anti-TNF and/or surgery (severe) (see Fig. 1). Clinical remission was defined by ulcerative colitis clinical score (UCCS) < 2 [29] and/or calprotectin level < 100 mg/kg according to Feagan et al. Faecal calprotectin was measured by an ELISA kit from Calpro Norway (Oslo, Norway).

### Tissue samples

Colonic mucosal biopsies were sampled from the region with the most severe inflammation. In healthy controls, biopsies were sampled from the sigmoid. Biopsy specimens for RNA extraction were immediately immersed in RNA *later* (Qiagen) and stored at room temperature overnight, then at − 20 °C until RNA isolation.

### Cytokine transcript measurements

Total RNA was isolated from patient biopsies using Trizol until July 1, 2008; later the Allprep DNA/RNA Mini Kit (Qiagen, Hilden, Germany, Cat No: 80204) and the automated QIAcube instrument (Qiagen, Hilden, Germany) according to the manufacturer’s recommendations. Quantity and purity of the extracted RNA were determined using the Qubit 3 Fluorometer (Cat No: Q33216; Invitrogen by Thermo Fisher Scientific, Waltham, MA, USA). Reverse transcription of the total RNA was performed using the QuantiTect Reverse Transcription Kit (Cat. No: 205314; Qiagen, Hilden, Germany). Mucosal TNF gene transcript was measured by real-time PCR procedures previously described in detail [30–33].

### Statistics

The following factors were evaluated as predictors: extent of disease, UCDAI score and endoscopic sub-score, histological activity score, fecal calprotectin and mucosal cytokine transcripts. All baseline predictors were standardized and centered for exploring combinations of two variables. To evaluate predictors of outcome, ROC curves were constructed. Optimal cut-off values were picked by maximal Youden’s J [34]. Test characteristics were derived by confusion matrices and diagnostic odds ratios [35]. A sequential test for mucosal TNF transcript and RHI score was constructed: Observations with a positive TNF test were run in a new ROC analysis for RHI score, which resulted in a two-step combined model with one cut-off value for mucosal TNF transcript and another cut-off value for RHI score following a positive TNF test.

As a global test, Kruskal Wallis one-way ANOVA was performed, then Mann-Whitney U test with Bonferroni correction. For categorical values Chi-square test with Bonferroni correction was utilized.

All statistical analyses were carried out in IBM SPSS Statistics 24 (IBM Corporation, Armonk, New York, USA).

## Results

### Healthy controls

Thirty-eight healthy controls were included, 13 females and 25 men aged 43–69 years. The median TNF value was 4450 copies/μg mRNA.

### Calibration cohort

#### Baseline characteristics and outcome groups

Sixty-six patients were included as a follow up mainly from an earlier report [19]. At 1 year follow-up patients were categorized into mild (*n* = 23), moderate (*n* = 18) and severe (*n* = 25) disease outcomes based on a step-up treatment level algorithm. In the moderate outcome group, no patients needed continuous steroid treatment and two patients were treated with azathioprine. In the severe outcome group, all patients were on anti-TNF treatment including one patient that later was in the need of colectomy. Sixteen patients were on concomitant treatment with azathioprine and one patient on methotrexate. An overview of baseline characteristics for each outcome group is shown in Table 1. There were significant differences between the three treatment groups for mucosal TNF and UCDAI scores (*p* < 0.017).

**Table 1:** Baseline characteristics of patients in the calibration cohort with ulcerative colitis according to one-year treatment outcome level

Patients groups	Mild *N* = 23	Moderate ***N*** = 18	Severe ***N*** = 25
Age med (IQR)	41 (35–54)	35 (24–55)	41 (27–54)
Sex			
Female	15 (65%)	7 (39%)	9 (36%)
Male	8 (35%)	11 (61%)	16 (64%)
Colonic area involved			
Proctitis	9 (39%)	3 (17%)	3 (12%)
Left side	9 (39%)	7 (39%)	10 (40%)
Extensive	5 (22%)	8 (44%)	12 (48%)
Smoking	14	21	12
Current smoker	4 (29%)	2 (17%)	2 (10%)
Non-smoker	10 (71%)	10 (83%)	18 (90%)
Mucosal TNF*	10,500 (4600–11,900)	12,000 (8000–17,200)	26,900 (18700–40,400)
UCDAI med (IQR)* at debut	7 (5–8)	9 (8–12)	12 (9–12)
Calprotectin med (IQR)	590 (400–1100)	790 (470–1540)	2300 (670–2500)
RHI med (IQR)	9 (5–10)	7 (6–10)	9 (7–12)
UCCS score 1-year med (IQR)	0 (0–0)	0 (0–2)	0 (0–2)
Calprotectin 1-year med (IQR)	60 (25–85)	50 (25–100)	25 (0–160)

*p < 0,017 between groups, Mann-Whitney U test with Bonferroni correction

*Med (IQR)* Median (Interquartile range), *RHI* Robarts histopathology index. Mucosal TNF in copies/μg RNA: Fecal calprotectin in mg/kg

#### Discovery of potential biomarkers

**Fig. 2:**
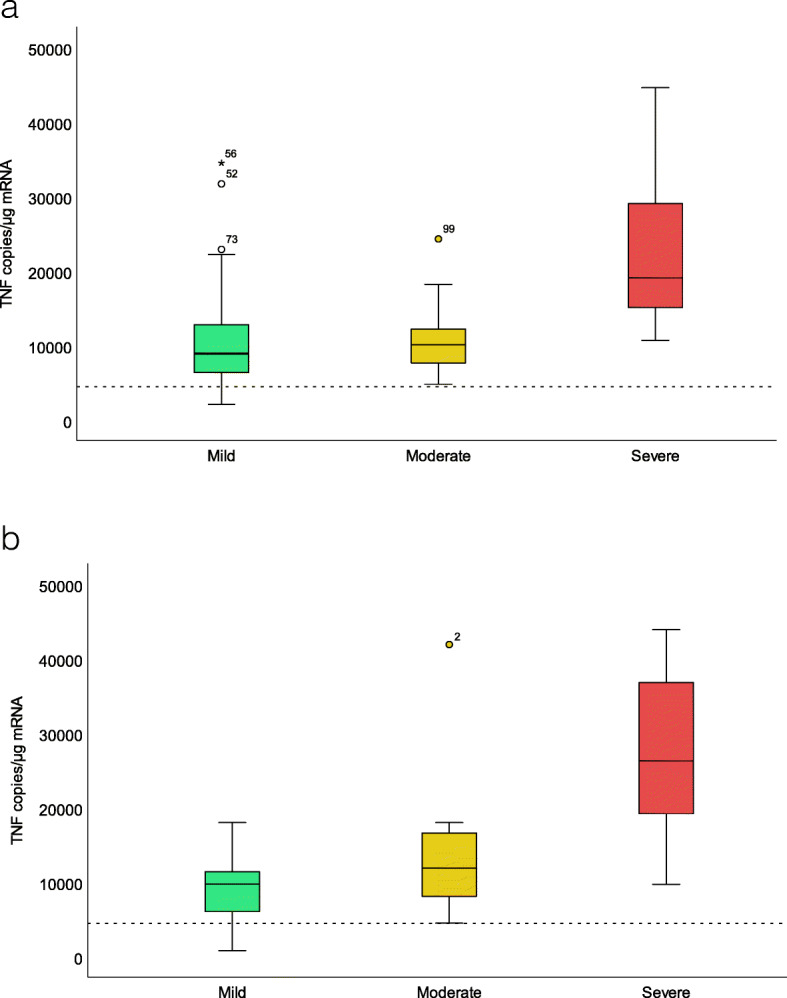
Mucosal TNF transcript in treatment outcome groups and in healthy normal controls in the calibration cohort (**a**) and the validation group (**b**)

With three defined treatment outcomes we made two sets of ROC curves, one set to discriminate between mild and moderate/severe and one set to discriminate between mild/moderate and severe. There were no baseline predictors that showed good test performance for discriminations between mild and moderate/severe (data not shown). However, there was a tendency towards increasing concentrations of the mucosal TNF transcripts with increasing treatment level (Fig. 2a).

#### Severe outcome

**Fig. 3:**
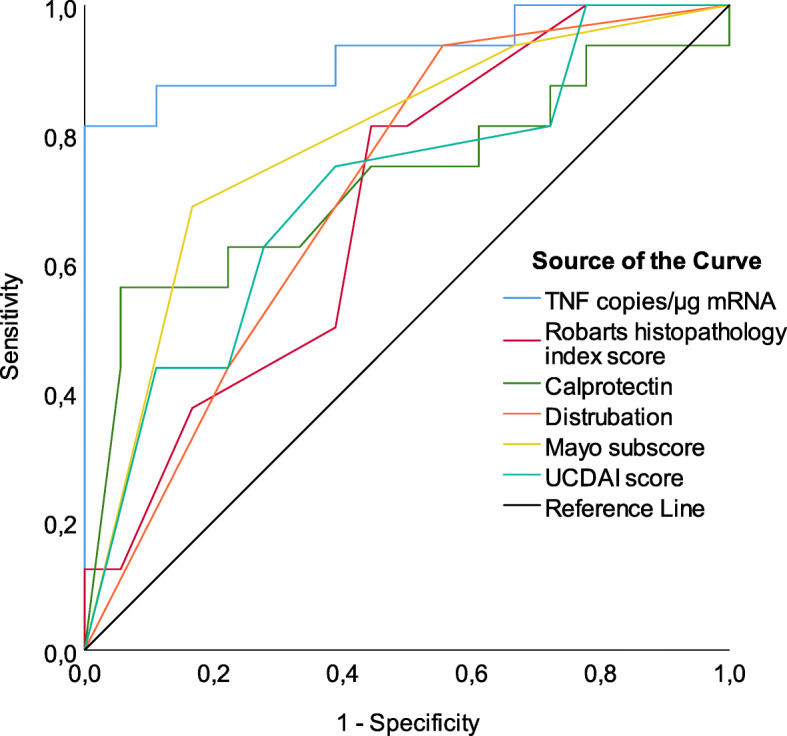
ROC curves of predictors of severe outcome in calibration cohorte

Baseline predictors of severe outcome are shown in Table 1 and Fig. 3 presenting clinical parameters (Calprotectin, UCDAI, Mayo endoscopic score), RHI score and mucosal TNF transcripts. Selected predictors including cut off values are shown in Table 3. Of individual factors, mucosal TNF transcript had the best test performance with a sensitivity, specificity and diagnostic odds ratio (DOR) of 0.81, 0.91 and 43 respectively. Clinical data including fecal calprotectin, UCDAI and RHI -score, yielded a high sensitivity but poor specificity (Table 2, Fig. 3), and therefore a poorer test performance than mucosal TNF transcript. To increase the test performance, we then combined mucosal TNF transcript and RHI score in a sequential setup: subjects with mucosal TNF transcript above cut-off were subjected to a second ROC curve using RHI score as predictor. The combined sequential test of mucosal TNF transcript and RHI score showed a superior test performance for specificity and DOR, however lower sensitivity (Table 2). No other clinical, biochemical, histological or immunological combinations could improve the test performance of prediction of severe outcome (supplement material Fig. 4).

**Table 2:** Factors at debut of ulcerative colitis in the calibration cohort to predict severe treatment outcomes at one year of disease

Factors	Youden’s J	Cut-off value	Sensitivity	Specificity	PPV	NPV	DOR
TNF^a^	0,72	≥18,000	0,81	0,91	0,85	0,89	43
RHI^a^	0,23	≥9	0,71	0,52	0,48	0,74	3
Combined TNF RHI	0,57	≥18,000 and ≥ 9	0,57	1	1	0,79	∞
UCDAI	0,4	≥9	0,79	0,61	0,54	0,83	6
Mayo subscore	0,45	3	0,72	0,73	0,62	0,81	7
Calprotectin	0,51	≥2000	0,6	0,91	0,86	0,72	15

Diagnostic odds ratio PPV: Positive predictive value *NPV* Negative predictive value

^a^ copies/μg mRNA ^b^Robarts histopathology index score

### Validation cohort

#### Baseline characteristics and outcome groups

At one year follow up patients were categorized into mild (*n* = 36), moderate (*n* = 31) and severe (*n* = 22) disease outcomes based on a step-up treatment level algorithm. In the moderate outcome group, no patients needed continuous steroid treatment and five subjects were treated with azathioprine. In the severe outcome group, 22 patients were on anti-TNF treatment whereas two of these patients were later in the need of colectomy. Thirty-eight healthy controls were included. An overview of baseline characteristics for each outcome group is shown in Table 3. There were significant differences between the three treatment groups for mucosal TNF, UCDAI, RHI scores and fecal calprotectin (Table 3, Fig. 2b).

**Table 3:** Baseline characteristics of patients with ulcerative colitis in the validation cohort according to one-year treatment outcome level

Patient groups	Mild ***N*** = 36	Moderate ***N*** = 31	Severe ***N*** = 22
Age med (IQR)	36 (24–49)	30 (24–41)	26 (22–47)
Sex			
Female	17 (47%)	8 (26%)	10 (46%)
Male	19 (53%)	23 (74%)	12 (54%)
Colonic area involved			
Proctitis	5 (14%)	1 (3%)	1 (4%)
Left side	25 (69%)	18 (58%)	10 (46%)
Extensive	6 (17%)	12 (39%)	11 (50%)
Smoking	28	21	12
Current smoker	1 (4%)	2 (10%)	1 (8%)
Non-smoker	27 (96%)	19 (90%)	11 (92%)
Mucosal TNF*	8800 (6100–12,800)	10,500 (7400–13,200)	17,400 (15100–26,800)
UCDAI med (IQR)* at debut	7 (5–9)	9 (8–11)	10 (7–11)
Calprotectin med (IQR)*	570 (200–970)	1000 (340–2000)	1100 (830–1400)
RHI med (IQR)*	6 (2–10)	6 (4–11)	14 (9–27)
UCCS score 1-year med (IQR)	0 (0–0)	0 (0–0)	0 (0–8)
Calprotectin 1-year med (IQR)	40 (25–94)	50 (0–140)	25 (20–60)

*p < 0,017 between groups, Mann-Whitney U test with Bonferroni correction

Med (IQR): median (Interquartile range); RHI: Robarts histopathology index. Mucosal TNF in copies/μg RNA; Fecal calprotectin in mg/kg

#### Validation of predictors of severe outcome

The cut off values from the discovery study (TNF ≥ 18,000, RHI ≥ 9) were used for test performance. The baseline predictors of severe outcome presenting mucosal TNF transcripts and RHI score are shown in Table 4. Mucosal TNF transcript had a test performance with sensitivity, specificity and DOR of 0.5, 0.9 and 9 respectively. RHI transcript had a test performance with sensitivity, specificity and DOR of 0.72, 0.69 and 6, respectively. When combined TNF and RHI the specificity increased to high 0.99, whereas the DOR was still high as 54. Moreover, the low sensitivity of 0.44 represents most likely the overlapping TNF and RHI score values to the mild/moderate outcome groups (Table 3).

**Table 4:** Factors at debut of ulcerative colitis in the validation cohort to predict severe treatment outcomes at one year of disease based on cut off values from the discovery cohort

Factors	Youden’s J	Cutt off value	Sensitivity	Specificity	PPV	NPV	DOR
TNF^a^	0,40	≥18,000	0,50	0,90	0,56	0,87	9
RHI^b^	0,41	≥9	0,72	0,69	0,38	0,90	6
Combined TNF RHI	0,43	18,000 ≥ 9	0,44	0,99	0,89	0,87	54

*DOR* Diagnostic odds ratio, *PPV* Positive predictive value *NPV* Negative predictive value

^a^ copies/μg mRNA ^b^Robarts histopathology index score

## Discussion

We present a combined discovery study (from 2004) and a validation study (from 2014) in a prospective design (the transomic Advanced Study of Inflammatory Bowel Disease) where clinical, biochemical, histological and transcript data where retrospectively tested to identify biomarkers of clinical outcome 1 year after disease diagnosis of UC. Mucosal TNF transcripts showed high test reliability for predicting severe outcome after 1 year in UC in both studies but was not ideal to discriminate between mild, moderate and severe disease. Moreover, when the TNF transcripts were combined with histological activity (RHI) scores, the test improved its diagnostic reliability. Mucosal cut-off values for TNF and RHI scores determined in the calibration cohort displayed a high test performance with specificity of 0.99 and a diagnostic odds-ratio (DOR) of 54 in the prospective validation study. Thus, mucosal TNF transcript combined with a histological score at debut of disease can likely identify patients who experience severe outcomes during the first year. This is an important step towards personalizing treatment in IBD and may be used as a criterion for selecting candidates for top-down treatment of anti-TNF. However, this awaits further studies.

We have tested a broad spectrum of potential factors that could, alone, or in combinations, predict clinical outcome in the first year of diagnosis. The clinical outcomes were defined as the highest treatment level required for achieving disease remission during the first year of disease, in a step-up treatment approach. The broad/wide selection of variables including various combinations did not have the necessary precision to discriminate between mild, moderate and severe outcomes. However mucosal TNF transcript in combination with the histological RHI score was able to predict, with high precision, the most severe colitis outcomes needing biological or surgical treatment, within the first year of disease. The validated cut-off values (TNF ≥18,000, RHI ≥ 9) showed a high specificity to predict severe outcome and a DOR as high as 54. From a clinical point of view, these cut–off values indicate a need of anti-TNF therapy during the first year after diagnosis with high reliability, and therefore of high clinical value and utility in the management of IBD/UC. In order to use a biomarker for selection for top-down treatment, a high PPV is necessary to avoid excessive use of biologics. Our proposed biomarker shows a PPV of 0.89 meaning that 9 out of 10 positives will be correctly identified as severe outcome.

A step-up treatment approach represents well-established international guidelines [8–10]. One drawback of this approach is that patients in the severe outcome group often experience a period of poor response during the gradual escalation of treatment intensity until an adequate response is obtained. In some cases, one may lose an important window of opportunity for optimal effect of biologics leading to permanent structural damage and/or need of surgery. The impact of early treatment before development of severe disease is not completely investigated. However, the top down approach published by D’Haens et al. indicated that immunosuppressive therapy was superior to a step-up approach in patients with Crohn’s disease [36]. Moreover, it is well documented that induction of treatment to remission reduces later hospitalization, whereas conflicting results exist for colectomy in two studies [37, 38].

The use of molecular data from the mucosa represents a novel approach and is an easily available tool, with high utility for clinicians to individually tailor therapy in UC. Endoscopic biopsies are routinely taken at diagnosis and surveillance of IBD. Thus, the logistics of measuring mucosal TNF transcript are simple, as biopsies are readily available and samples do not require freezing prior to analysis [31].

Our study contributes with new knowledge in the scientific field of personalized therapy in UC [15, 16]. We know that treatment to remission improves long-term clinical outcome [39, 40]. The main question is: Can a top-down therapy of the most severe forms of disease have an effect on the natural course of disease? This awaits future studies.

The strength of this prospective designed, combined discovery and validation study is that we have retrospectively searched for and validated biomarkers for treatment at debut of UC, using a broad search of clinical, histological and analytical factors including mucosal immune transcripts. Moreover, this is part of the transomic Advanced Study of Inflammatory Bowel disease (ASIB) study where parallel studies of the epigenome, transcriptome, proteome and metabolome are ongoing [33, 41–45]. .This transomic approach at debut of UC will be performed and correlated to long-term clinical outcome. Therefore, the upcoming transomic data from the ASIB study and from several ongoing studies such as the PREDICTS study will not only search for therapeutic but also prognostic and natural course biomarkers [17]. The weakness of the study includes the lack of endoscopic diagnosis at 1 year, which would have given insight into endoscopic status and endoscopic remission rates according to treatment levels. Additionally, the decision to use or not use steroids at time of diagnosis is dependent on the subjective decision of the clinicians. This may be one explanation for the small differences detected between the mild and moderate treatment group.

## Conclusion

The combined information of mucosal TNF transcription and histological score at debut of UC can predict severe outcome and the need for anti-TNF therapy. This is of great clinical utility and may contribute to a personalized medicine approach in UC.

## Supplementary information

**Additional file 1: Figure 4.** Supplement figure with ROC curves of predictors of mild outcome from calibration cohort

## Data Availability

Data are available from the authors upon reasonable request due to privacy/ethical restrictions.

## Abbreviations

RHI: Robarts histopathology index
DOR: Diagnostic odds-ratio
UC: Ulcerative colitis
IBD: Inflammatory bowel disease
TNF: Tumor necrosis factor
ASIB- study: Advanced Study of Inflammatory Bowel disease
UCCS: Ulcerative colitis clinical score
