# Supplementary figures and images for: Discovery and validation of mucosal TNF expression combined with histological score - a biomarker for personalized treatment in ulcerative colitis
Source: BMC Gastroenterol. 2020 Oct 2;20:321. doi: 10.1186/s12876-020-01447-0 (PMC7532085; doi:10.1186/s12876-020-01447-0)

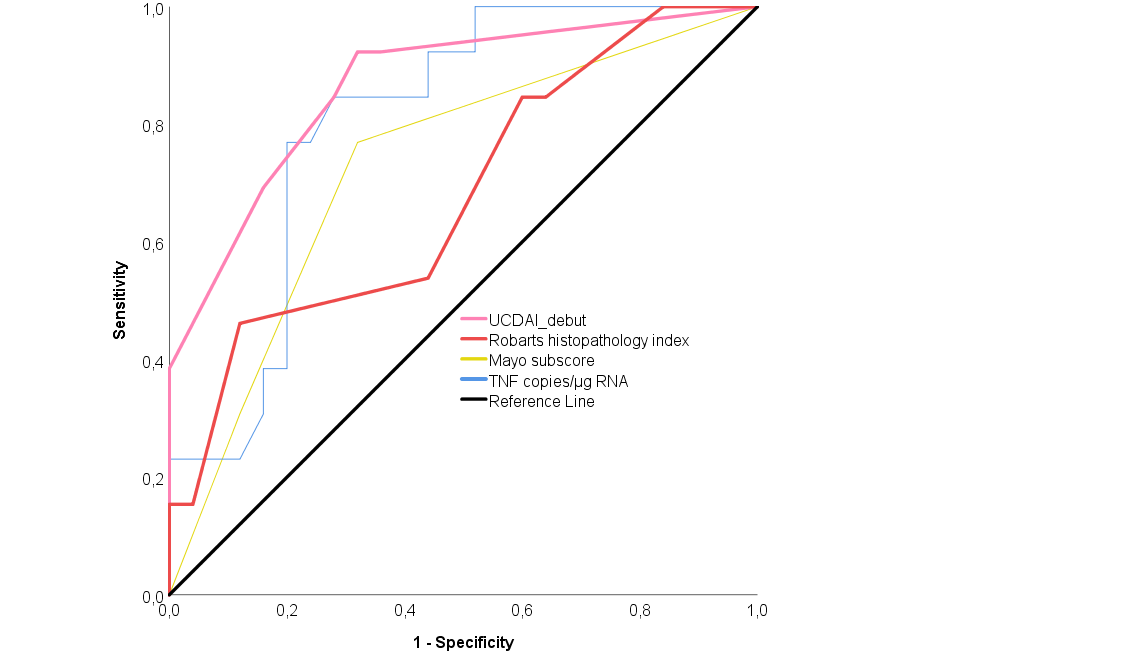

Supplement: Supplementary file 1 — Additional file 1: Figure 4. Supplement figure with ROC curves of predictors of mild outcome from calibration cohort [file 12876_2020_1447_MOESM1_ESM.tif]
